# Supplementary material for: Nuclear-capture of endosomes depletes nuclear G-actin to promote SRF/MRTF activation and cancer cell invasion
Source: Nat Commun. 2021 Nov 24;12:6829. doi: 10.1038/s41467-021-26839-y (PMC8613289; doi:10.1038/s41467-021-26839-y)
Supplement: Supplementary file 1 — Supplementary Information [file 41467_2021_26839_MOESM1_ESM.pdf]

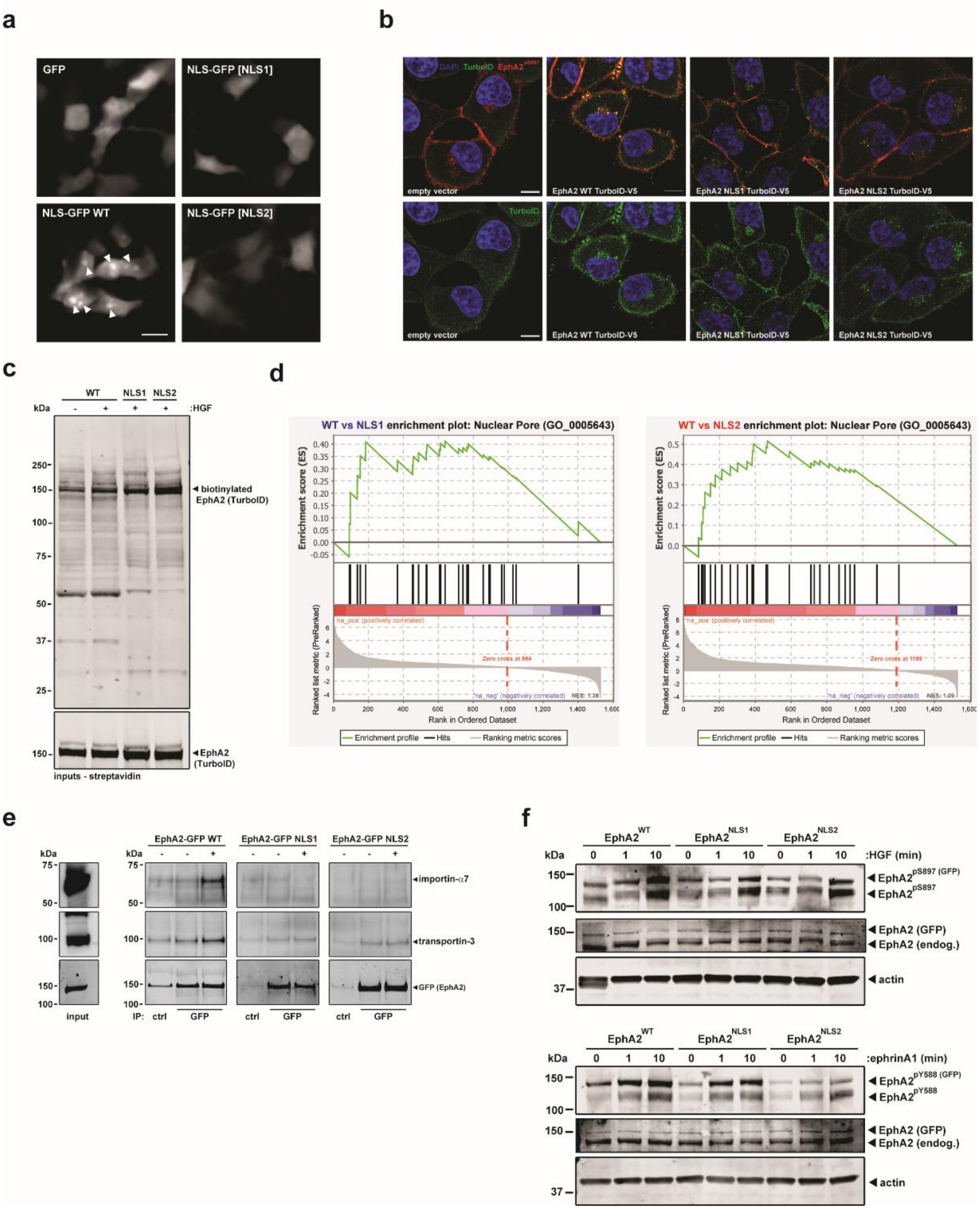

Supplementary figure 1: EphA2's NLS and its role in interaction with the nuclear import machinery

a. H1299 cells were transfected with constructs encoding GFP, GFP fused to a sequence corresponding to the putative nuclear localisation signal from the juxtamembrane region of EphA2 (NLS-GFP WT) or EphA's NLS with the alanine substitutions indicated in figure 3a (NLS-GFP [NLS1] and NLS2-GFP [NLS2]). Cells were imaged using fluorescence confocal microscopy. Bar, 20 µm. Arrows indicate nuclear localisation of GFP.

b. Cells expressing EphA2-TurboID constructs for wild-type or EphA2 NLS mutants were stained with V5-Tag and EphA2 (p-Ser^{897}) antibodies. Cells were imaged using fluorescence confocal microscopy. Bar, 10 µm.

c. Cells stably expressing the indicated EphA2-TurboID constructs were incubated with biotin with or without HGF for 1 hr. The biotin ligation efficiency of the TurboID construct was assessed by Western blotting with fluorophore-conjugated streptavidin.

d. The plots show the GSEA enrichment of nuclear pore components based on the data obtained in the proteomics mass spectrometry analysis presented in Fig.3c. Hits to the left of the dotted red line are those which display greater biotinylation with WT than they do with NLS mutated TurboIDs.

e. Cells expressing EphA2-GFP, EphA2 NLS1-GFP or EphA2 NLS2-GFP were treated with HGF for 5 min. EphA2-GFPs immunoprecipitated and importin–α7 and tranportin-3 in the immunoprecipitates was determined by Western blotting.

f. Cells were transfected with either wild-type EphA2 ($EphA2^{WT}$) or EphA2s with mutations in its nuclear localisation sequence ($EphA2^{NLS1}$ and $EphA2^{NLS2}$). Transfected cells were treated with HGF (left panels) or ephrinA1-Fc ligand (right panels). Phosphorylation of EphA2 at Ser^{897} (left panels) and Tyr^{588} (right panels) was determined by Western blotting.

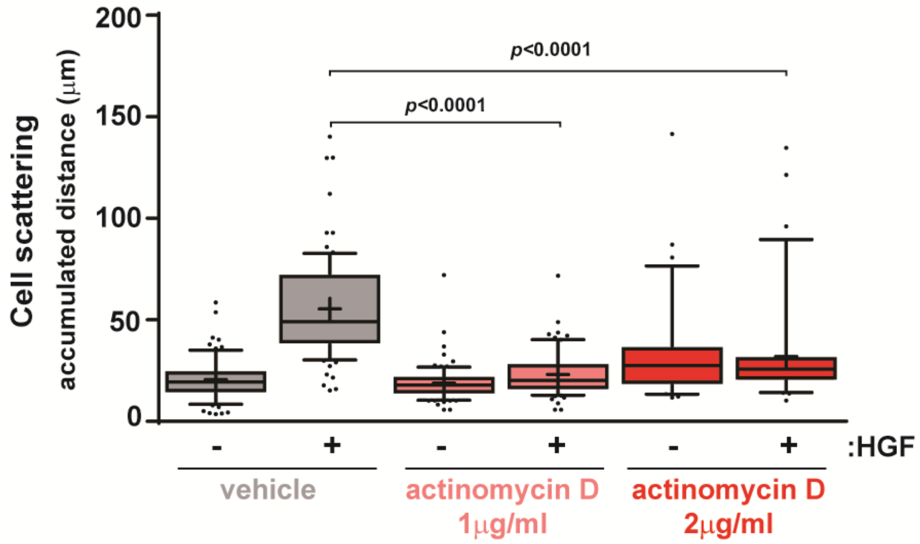

Supplementary figure 2: Transcription is necessary for cell scattering

H1299 cells were plated onto glass surfaces and allowed to form colonies of approx. 4 cells/colony for 48h. Then, cells were treated with vehicle, or actinomycin D (an inhibitor of DNA transcription initiation) with either 1 or 2µg/ml, and 30min later cells were challenged with HGF as indicated. Cell scattering was quantified using ImageJ and is expressed as the accumulated distance travelled over 8 hr. Box and whiskers: 10-90 percentile whiskers, + represents mean, black line represents median, n=3 independent experiments, statistical test is one-way ANOVA.

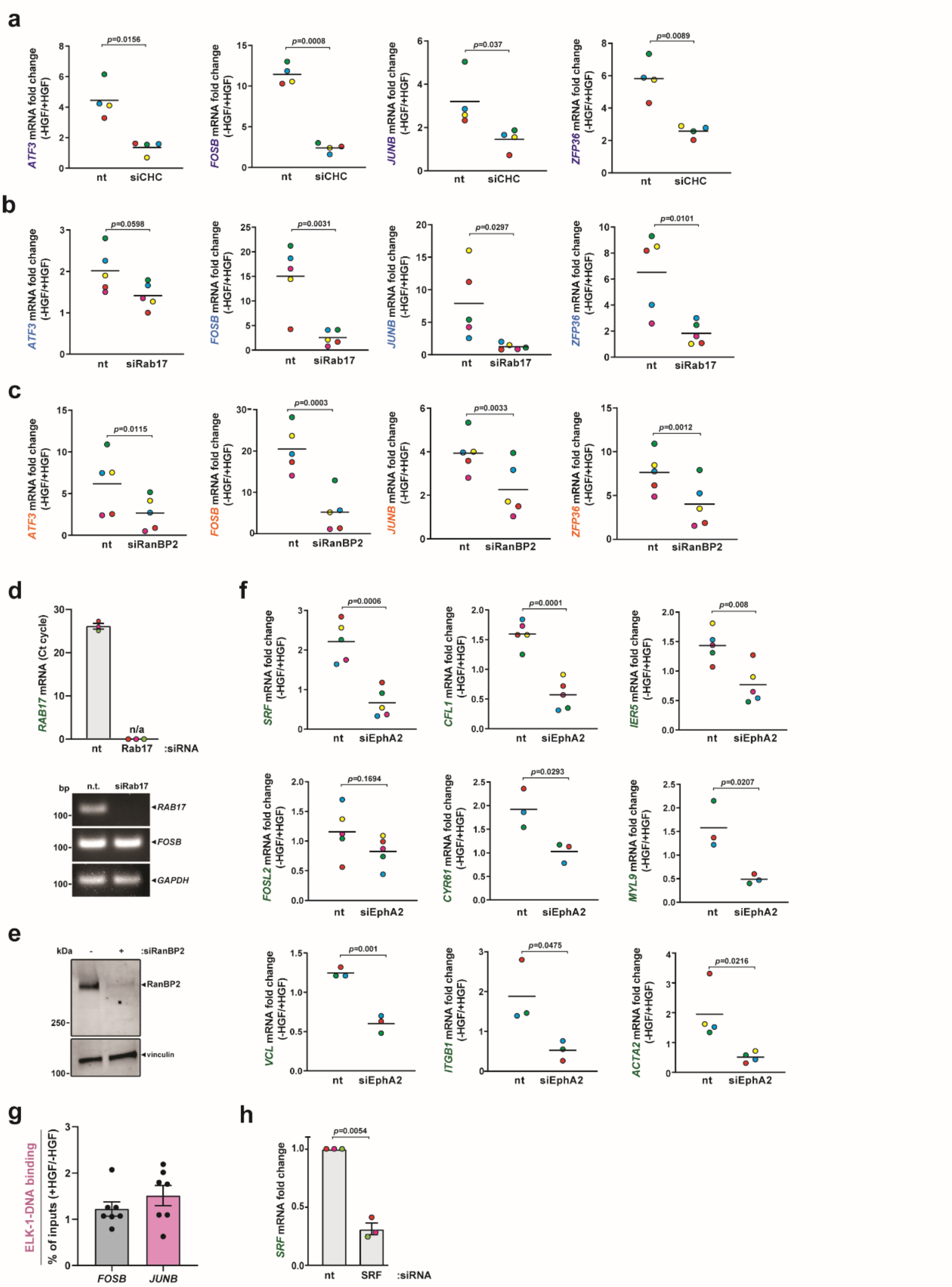

Supplementary figure 3: The role of nuclear-capture expression of MRTF/SRF target genes

a-c. H1299 cells were transfected with siRNAs targeting Clathrin Heavy Chain (siCHC), Rab17 (siRab17), RanBP2 (siRanBP2) or non-targeting control (nt), treated with HGF and the HGF-induced change in the indicated mRNAs (*FOSB; JUNB; ATF3; ZFP36*) determined using

qPCR. Bars are the means of 4 (a) or 5 (b,c) independent experiments, statistical test is paired t-test two-sided. Paired data are denoted by dots of the same colour.

d-e Cells were transfected with siRNAs targeting Rab17 (siRab17), RanBP2 (siRanBP2) or non-targeting control (nt). Knockdown of Rab17 was determined using qPCR and knockdown of RanBP2 was determined by Western blotting using vinculin as loading control (e). The lower panel in (d) corresponds to electrophoretic analysis of the Rab17 PCR products.

f. Cells were transfected with siRNA targeting EphA2 (siEphA2) or non-targeting control (nt). Cells were challenged with HGF and the HGF-induced change in levels of the indicated mRNAs (*FOSB; JUNB; ATF3; ZFP36; SRF; CFL1, IER5, FOSL2; CYR61; MYL9; VCL; ITGB1; ACTA2*) determined as for (a). Bars are means of 5 (*SRF, CFL1, IER5, FOSL2*), 4 (*ACTA2*) or 3 (*CYR61, MYL9, VCL, ITGB1*) independent experiments. Statistical test is t-test two-sided. Paired data are denoted by dots of the same colour.

g. Cells were incubated in the presence or absence of HGF for 20 min and the DNA sheared by sonication. Elk-1 was immunoprecipitated from lysates and the quantity of the indicated genes (*FOSB; JUNB*) determined using qPCR. The HGF-induced fold change in immunoprecipitated genes is plotted on the y-axis. Values are mean ± sem, n=6 independent experiments.

h. Cells were transfected with siRNAs targeting SRF (siSRF) or non-targeting control (nt). Knockdown of SRF was determined using qPCR. Values are mean ± sem, n=3 independent experiments, statistical test two-sided paired t-test.

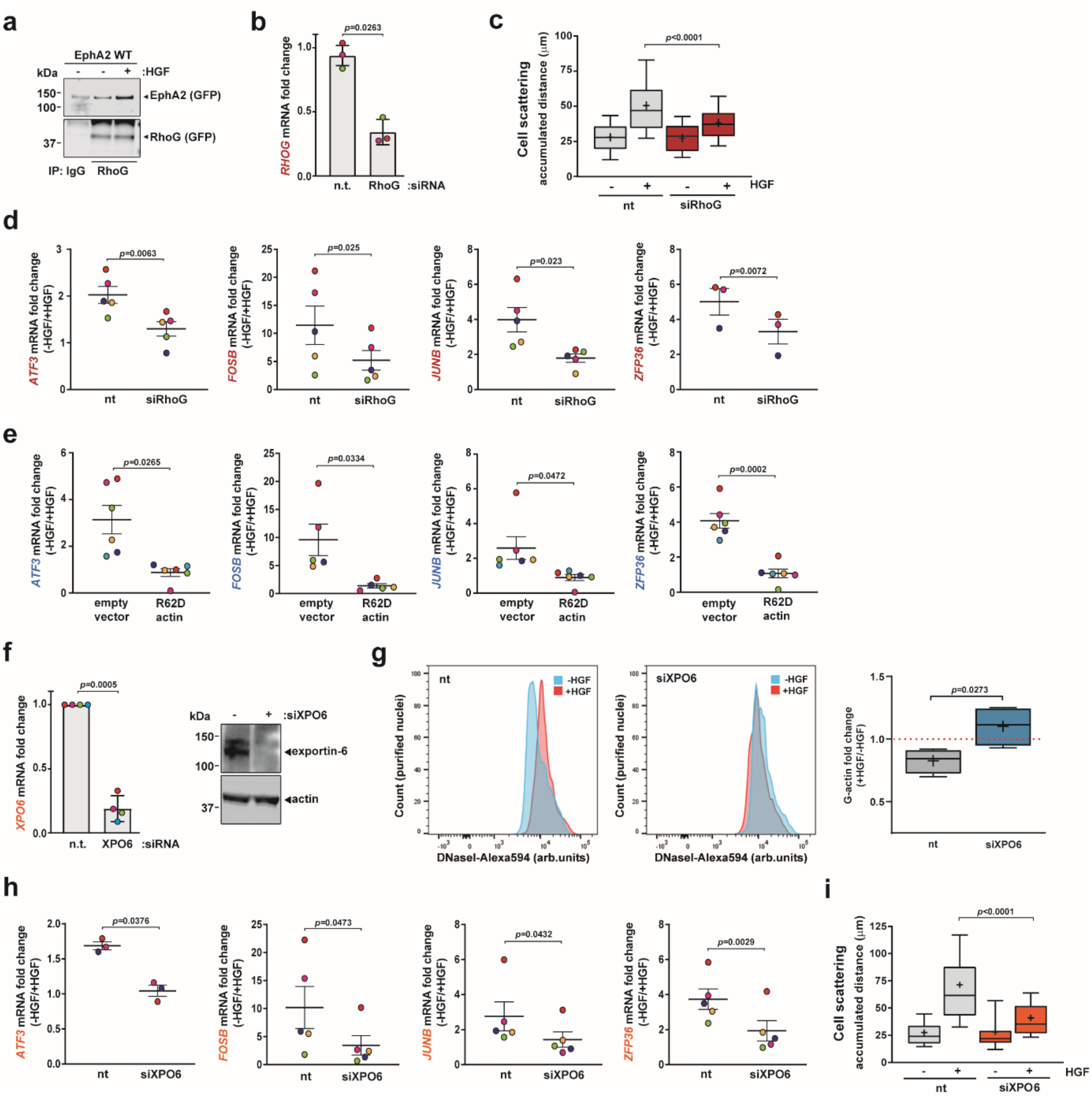

Supplementary figure 4: Nuclear G-actin dynamics and MRTF/SRF target gene expression

a. H1299 cells were transfected with GFP-RhoG in combination with EphA2-GFP and incubated in the presence or absence of HGF for 20 min. Cells were lysed, immunoprecipitated (IP) using magnetic beads coupled to an antibody recognising RhoG or a non-specific control antibody (IgG). The presence of RhoG and EphA2 in the immunoprecipitates was determined by Western blotting.

b-e. H1299 cells were transfected with an siRNA targeting RhoG (siRhoG) or non-targeting control (nt) (b-d) or an expression vector encoding a non-polymerisable mutant of actin (R62D actin) or vector control (empty vector) (e). The efficiency of the knockdown on RhoG was assessed by qPCR (b). In (c), cell scattering in the presence and absence of HGF was determined as for Fig. 4f. n=3 independent experiments, statistical test is one-way ANOVA. In

(d-e) cells were challenged with HGF or were left unchallenged for 20 min and levels of the indicated mRNAs were determined using qPCR. Bars are mean±sem ([d] n=5 *ATF3, FOSB, JUNB, n=3 ZFP36*; [e] n=6 *ATF3, ZFP36, JUNB,* n=5 *FOSB*; independent experiments), statistical test is paired t-test. Paired data are denoted by dots of the same colour.

f-i. Cells were transfected with an siRNA targeting exportin-6 (siXPO6) or non-targeting control (nt). Knockdown of *XPO6* was assessed by qPCR and Western blotting (f). Cells were challenged with HGF for 20 min and levels of G-actin in purified nuclei determined using flow cytometry as for Fig. 6c (g), and expression of the indicated MRTF/SRF target genes determined using qPCR (h). Bars are mean±sem of 5 (*FOSB, JUNB, ZFP36*) or 3 (*ATF3*) independent experiments, statistical test is paired t-test. Paired data are denoted by dots of the same colour. In (i) cell scattering in the presence and absence of HGF was measured as for Fig. 4f. n=3 independent experiments, statistical test is one-way ANOVA.

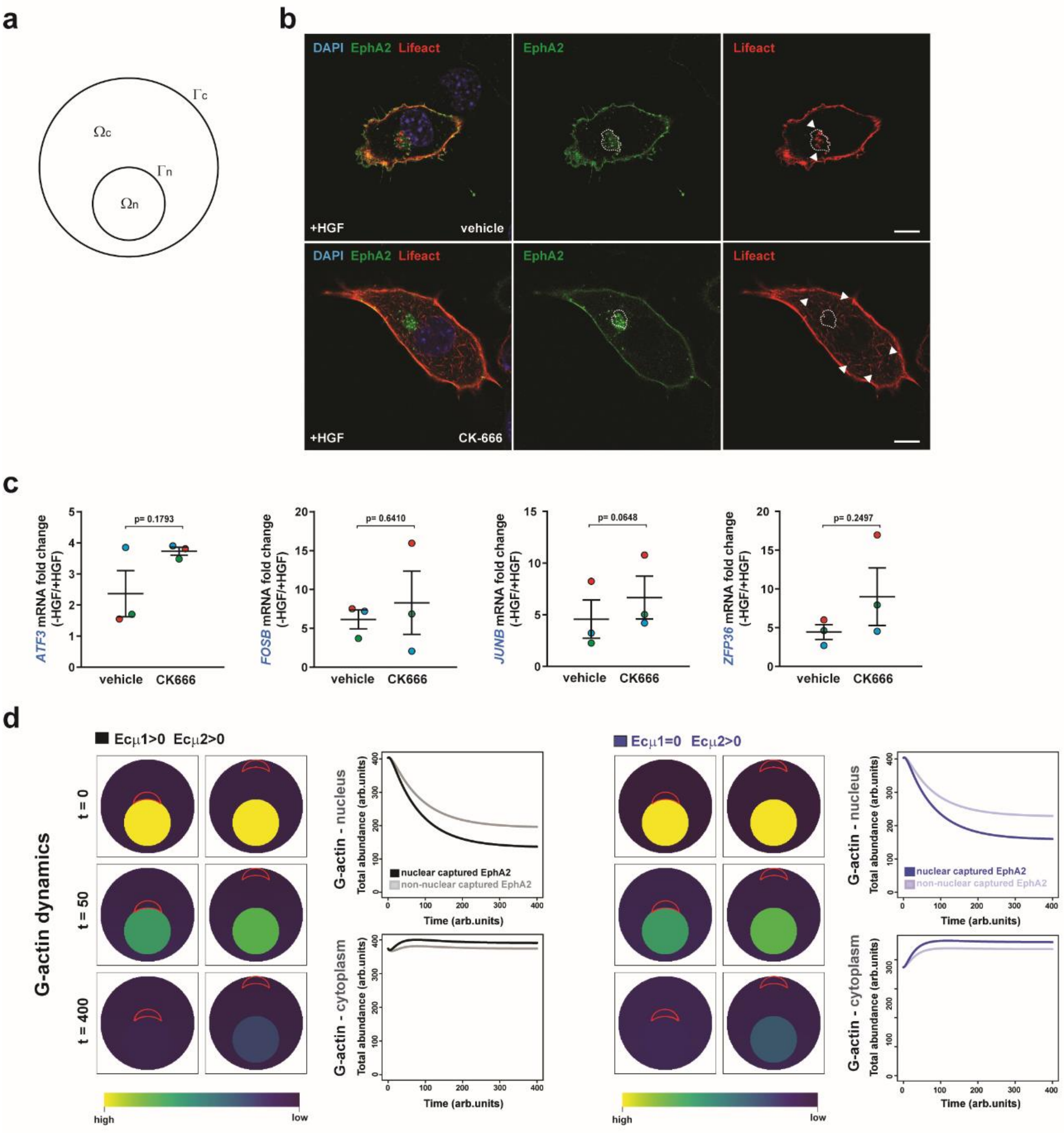

Supplementary figure 5: Inhibition of juxta-nuclear actin polymerisation does not oppose HGF-driven expression of MRTF/SRF target genes

a. Graphical illustration of the simulation domains representing the cytoplasmic and nuclear compartments. The cell is modelled as two static circular compartments Ω_c and Ω_n, which represent the cytoplasm and the nucleus, respectively. Γ_c and Γ_n denote the external boundary of the cytoplasm and the nucleus:cytoplasm boundary respectively

b. H1299 cells were transfected with wild-type EphA2-GFP (EphA2; green) in combination with mCherry-Lifeact (Lifeact; red). Transfected cells were incubated in the presence of the Arp2/3 inhibitor, CK-666 or vehicle control. Cells were monitored using high resolution time-lapse fluorescence imaging following challenge with HGF and movies collected. Stills (captured 20 min following HGF addition) from these movies are presented. Bar, 10 µm. The juxta-nuclear regions are delineated with dotted lines.

c. H1299 cells were incubated in the presence or absence (vehicle) of the Arp2/3 inhibitor, CK-666 and then challenged with HGF for 20 min. HGF driven expression of the indicated MRTF/SRF target genes was determined using PCR as for figure 5b. Each bar is the mean of 3 independent experiments ± sem, P values as determined using paired t-test.

d. The same computational model as in Fig. 7c was used. Here, the images depict a modelled cell in which the area of influence of EphA2 is delimited by a red lined crescent, which now is located next to the boundaries of the cell, thus simulating receptor localisation to the the plasma membrane. The influence of increasing the rates of actin polymerisation ($E_c\mu1$) and cofilin phosphorylation ($E_c\mu2$) on depletion of nuclear G-actin was evaluated. The left-hand images depict predicted alterations to G-actin distribution following activation of both juxta-nuclear actin polymerisation and cofilin phosphorylation effected by increasing the rates $E_c\mu1$ and $E_c\mu2$ to values greater than zero ($E_c\mu1>0$ and $E_c\mu2>0$). The centre image panels depict alterations to G-actin distribution occurring following activation of actin polymerisation ($E_c\mu1>0$), but not cofilin phosphorylation ($E_c\mu2=0$). The right-hand image panels depict predicted G-actin dynamics following activation of cofilin phosphorylation ($E_c\mu2>0$), but not actin polymerisation ($E_c\mu1=0$). The dynamics of G-actin either in the nucleus (top graph) or in the cytoplasm (bottom graph), and the final values for nuclear G-actin levels (intensity scale) are represented for either nuclear-captured EphA2 activity (grey line and grey arrowhead respectively) or non-nuclear captured EphA2 activity (black line and black arrowhead respectively) conditions.

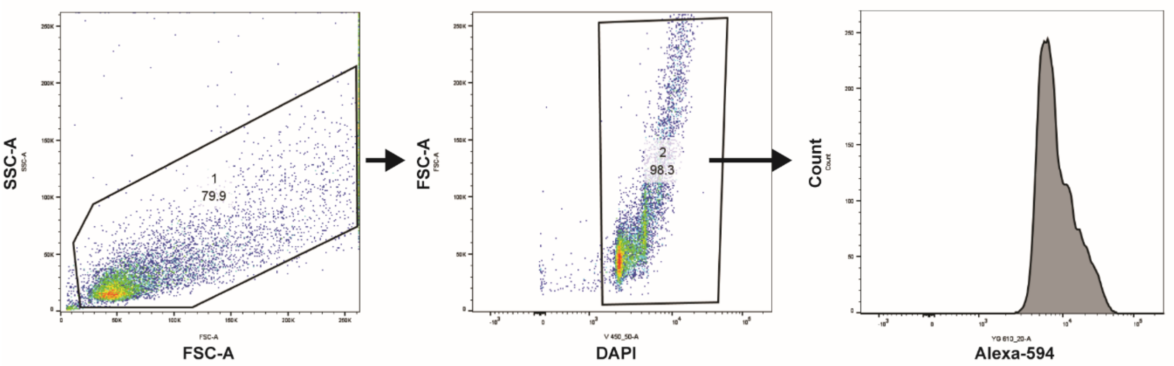

Supplementary figure 6: Gating strategy for G-actin quantification in purified nuclei

This Supplementary figure is related to Fig. 6c and Supplementary figure 4g.

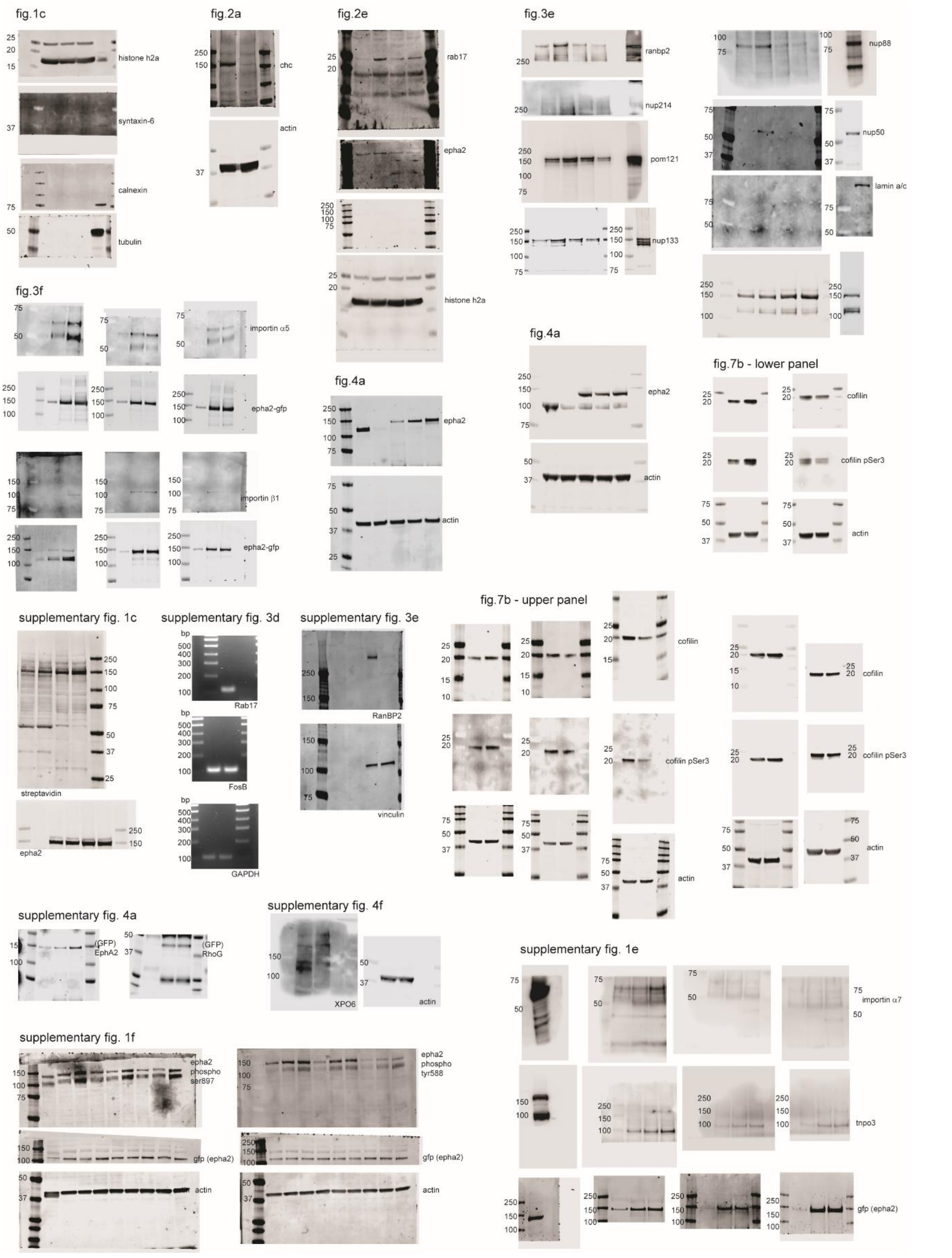

Supplementary figure 7: Uncropped scans of Western blots

Molecular weights are in kDa, except for figure 3d.

JunB mouse	F – 5’- TCA CGA CGA CTC TTA CGC AG -3’<br>R – 5’- CCT TGA GAC CCC GAT AGG GA – 3’
FosB mouse	F – 5’- TTT TCC CGG AGA CTA CGA CTC -3’<br>R – 5’- GTG ATT GCG GTG ACC GTT G – 3’
ATF3 mouse	F – 5’-GAG GAT TTT GCT AAC CTG ACA CC -3’<br>R – 5’- TTG ACG GTA ACT GAC TCC AGC – 3’
Zfp36 mouse	F – 5’- CCA CCT CCT CTC GAT ACA AGA -3’<br>R – 5’- GCT TGG CGA AGT TCA CCC A – 3’
ZFP36 human	F – 5’- GAC TGA GCT ATG TCG GAC CTT -3’<br>R – 5’- GAG TTC CGT CTT GTA TTT GGG G – 3’
SRF human	F - 5’- ACT GCC TTC AGT AGG AAC AA - 3’<br>R – 5’- TTC AAG CAC ACA CAC TCA CT – 3’
ACTA2 human	F- 5’ – AAA AGA CAG CTA CGT GGG TGA - 3’<br>R – 5’ – GCCATGTTCTATCGGGTACTTC – 3’
CFL1 human	F – 5’ – TAC GCC ACC TTT GTC AAG ATG -3’<br>R – 5’ - CCT TGG AGC TGG CAT AAA TCA T - 3’
IER5 human	F – 5’ – TTT CTC GGG ACT CCT ACG GAA – 3’<br>R – 5’ - GCT CCA GGG GTT CAT GTC TC – 3’
FOSL2 human	F – 5’ – CAG AAA TTC CGG GTA GAT ATG CC – 3’<br>R – 5’ – GGT ATG GGT TGG ACA TGG AGG – 3’
CYR61 human	F – 5’ – CTC GCC TTA GTC GTC ACC C – 3’<br>R – 5’ - CGC CGA AGT TGC ATT CCA G – 3’
MYL9 human	F – 5’ – TCT TCG CAA TGT TTG ACC AGT – 3’<br>R – 5’ – GTT GAA AGC CTC CTT AAA CTC CT – 3’
VCL human	F – 5’ – CCA AGA TGA TTG ACG AGA GAC AG – 3’<br>R – 5’ – AGA GGT GAG TTG TAA CAC ACG A – 3’
ITGB1 human	F- 5’ – CCT ACT TCT GCA CGA TGT GAT G– 3’<br>R- 5’ – CCT TTG CTA CGG TTG GTT ACA TT – 3’
ARHGEF16 human	F- 5’ – ATC TGG ACG CTG TTT TTG GAC – 3’<br>R- 5’ – GGC ATC TTC GTA CCT GTT GAG A – 3’
18S ribosomal	F- 5’ – GCT TAA TTT GAC TCA ACA CGG GA – 3’<br>R- 5’ – AGC TAT CAA TCT GTC AAT CCT GTC – 3’
XPO6 human	F- 5’ – ATC TGG ACG CTG TTT TTG GAC – 3’<br>R- 5’ – GGC ATC TTC GTA CCT GTT GAG A – 3’
RANBP2 human	F- 5’ – AAA CCT CCG ATT GCA GCT CAT – 3’<br>R- 5’ – GGC AAA GAT GGC CTT AAT CCT – 3’
FOSB promoter human	F- 5’ – CCG CGA GCA GTT CCC GTC AAT CCC TC - 3’<br>R- 5’ – GCA GTT CCT GTC TCA GAG GTC TCG TGG GC - 3’
JUNB promoter human	F- 5’ – CCT CCC GGG TCC CTG CAT CCC C – 3’<br>R- 5’ – ACG CCT CTC GGC CCT CTC TTC CC – 3’
ATF3 human	Qiagen Cat. No. QT00000273
FOSB human	Qiagen Cat. No. QT00013076
JUNB human	Qiagen Cat. No. QT00201341
ARHGEF16 human	Qiagen Cat. No. QT00229453
RAB17 human	Qiagen Cat. No. QT00009590

Supplementary table 1. Sequences of primers used for PCR

Marco et al., Supplementary table 2. Default simulation parameters for prediction of nuclear G-actin dynamics

Quantity	Symbol	Value
Centre of the nucleus	$\boldsymbol{p}_1$	$(0, -0.35)$
Centre of the cell	$\boldsymbol{p}_2$	$(0,0)$
Centre of the catalytic growth factor	$\boldsymbol{p}_3$	$(0,0)$
Radius of the nucleus	r_1	5×10^{-1}
Radius of the cell	r_2	1
Radius of the catalytic growth factor	r_3	3×10^{-1}
Diffusion coefficient of G-actin in the cytoplasm	D_{G_c}	1×10^{-1}
Diffusion coefficient of F-actin in the cytoplasm	D_{F_c}	0
Diffusion coefficient of cofilin in the cytoplasm	D_{C_c}	1×10^{-1}
Diffusion coefficient of cofilin-actin in the cytoplasm	D_{Ξ_c}	1×10^{-1}
Diffusion coefficient of profilin in the cytoplasm	D_{P_c}	1×10^{-1}
Diffusion coefficient of profilin-actin in the cytoplasm	D_{Υ_c}	1×10^{-1}
Diffusion coefficient of phospho-cofilin in the cytoplasm	D_{Θ_c}	1×10^{-1}
Diffusion coefficient of catalytic growth factor in the cytoplasm	D_{E_c}	0
Diffusion coefficient of G-actin in the nucleus	D_{G_n}	1×10^{1}
Diffusion coefficient of cofilin in the nucleus	D_{C_n}	1×10^{1}
Diffusion coefficient of cofilin-actin in the nucleus	D_{Ξ_n}	1×10^{1}
Diffusion coefficient of profilin in the nucleus	D_{P_n}	1×10^{1}
Diffusion coefficient of profilin-actin in the nucleus	D_{Υ_n}	1×10^{1}
Rate of polymerisation for G-actin in the cytoplasm	γ_{G_c}	2×10^{-2}
Rate of depolymerisation for F-actin in the cytoplasm	γ_{F_c}	1×10^{-2}
Catalytic rate parameter for actin polymerisation	μ_1	1
Rate of association between G-actin and cofilin in the cytoplasm	α_{Ξ_c}	1×10^{-1}
Rate of dissociation for cofilin-actin in the cytoplasm	γ_{Ξ_c}	1×10^{-2}
Rate of association between G-actin and profilin in the cytoplasm	α_{Υ_c}	1×10^{-2}
Rate of dissociation for profilin-actin in the cytoplasm	γ_{Υ_c}	1
Rate of phosphorylation for cofilin in the cytoplasm	α_{Θ_c}	2×10^{-2}
Rate of dephosphorylation for phospho-cofilin in the cytoplasm	γ_{Θ_c}	4×10^{-2}
Catalytic rate parameter for cofilin phosphorylation	μ_2	5×10^{1}
Decay rate of catalytic growth factor	γ_{E_c}	0
Rate of association between G-actin and cofilin in the nucleus	α_{Ξ_n}	1×10^{-2}

Marco et al., Supplementary table 2. Default simulation parameters for prediction of nuclear G-actin dynamics

Rate of dissociation for cofilin-actin in the nucleus	γ_{Ξ_n}	1
Rate of association between G-actin and profilin in the nucleus	α_{Υ_n}	1×10^{-1}
Rate of dissociation for profilin-actin in the nucleus	γ_{Υ_n}	1×10^{-2}
Nuclear import rate of profilin	β_{P_c}	1
Nuclear export rate of profilin	β_{P_n}	1
Nuclear export rate of profilin-actin	β_{Υ_n}	1
Nuclear import rate of cofilin	β_{C_c}	1
Nuclear export rate of cofilin	β_{C_n}	1
Nuclear import rate of cofilin-actin	β_{Ξ_c}	2

Marco et al., Supplementary table 3. Initial computed values for species used for simulations

Quantity	Symbol	Value
Initial concentration of G-actin in the cytoplasm	$G_{c_{\text{init}}}$	2.64×10^{-1}
Initial concentration of F-actin in the cytoplasm	$F_{c_{\text{init}}}$	5.28×10^{-1}
Initial concentration of catalytic growth factor	$E_{c_{\text{init}}}$	10
Initial concentration of cofilin in the cytoplasm	$C_{c_{\text{init}}}$	1.40×10^{-1}
Initial concentration of cofilin-actin in the cytoplasm	$\Xi_{c_{\text{init}}}$	9.12×10^{-3}
Initial concentration of profilin in the cytoplasm	$P_{c_{\text{init}}}$	1.34×10^{-1}
Initial concentration of profilin-actin in the cytoplasm	$\Upsilon_{c_{\text{init}}}$	3.95×10^{-3}
Initial concentration of phospho-cofilin in the cytoplasm	$\Theta_{c_{\text{init}}}$	6.99×10^{-2}
Initial concentration of G-actin in the nucleus	$G_{n_{\text{init}}}$	8.56×10^{-1}
Initial concentration of cofilin in the nucleus	$C_{n_{\text{init}}}$	1.50×10^{-1}
Initial concentration of cofilin-actin in the nucleus	$\Xi_{n_{\text{init}}}$	1.21×10^{-2}
Initial concentration of profilin in the nucleus	$P_{n_{\text{init}}}$	1.27×10^{-1}
Initial concentration of profilin-actin in the nucleus	$\Upsilon_{n_{\text{init}}}$	2.74×10^{-3}
