# Supplementary material for: Nuclear-capture of endosomes depletes nuclear G-actin to promote SRF/MRTF activation and cancer cell invasion
Source: Nat Commun. 2021 Nov 24;12:6829. doi: 10.1038/s41467-021-26839-y (PMC8613289; doi:10.1038/s41467-021-26839-y)
Supplement: Supplementary file 3 — Description of Supplementary Files [file 41467_2021_26839_MOESM3_ESM.docx]

**Legends to supplementary movies**

**Supplementary movie 1: Internalisation and centripetal transport of EphA2/Rab17-positive endosomes.** H1299 cells were transfected with EphA2-GFP (green) in combination with Rab17-mCherry (red). Transfected cells were challenged with HGF and monitored using confocal time-lapse fluorescence imaging. Arrows indicate EphA2/Rab17-positive vesicles that move centripetally from the cell’s periphery to the juxta-nuclear region.

**Supplementary movies 2 & 3: Interaction of EphA2-positive vesicles with α5 and β1 importins in the perinuclear region.**  H1299 cells were transfected with EphA2-GFP (green) in combination with mCherry- importin-α5 (red channel; movie 2) or mCherry-importin-β1 (red channel, movie 3), imaged by fluorescence time-lapse microscopy and movies collected. Arrows indicate where EphA2 and importin-positive structures come into contact for an extended period.

**Supplementary movies 4 – 6:** H1299 cells were transfected with wild-type EphA2-GFP (WT; green signal movies 4 & 6) or an NLS mutant of EphA2-GFP (NLS2; green signal movie 5) in combination with mCherry-Lifeact (red signal) in the presence (movie 6) or absence (movies 4 & 5) of an siRNA targeting RhoG (siRhoG). Cells were challenged with HGF at the start of the movie and monitored using high resolution time-lapse fluorescence imaging. Arrows indicate events in which actin polymerisation was observed to increase in the vicinity of an EphA2-positive vesicle.

**Supplementary movie 7:** A computational approach was used to model nucleocytoplasmic actin dynamics according to the schematic in Fig. 4a. The movies depict model cells in which the nuclear envelope is clearly shown and the alterations to G-actin levels are represented by the Viridis colour palette. The influence of altering the rates of actin polymerisation (E_c_µ1) and cofilin phosphorylation (E_c_µ2) on depletion of nuclear G-actin was evaluated. The left-hand movie and graph (black line) depict predicted alterations to G-actin distribution following activation of both juxtanuclear actin polymerisation and cofilin phosphorylation effected by increasing the rates E_c_µ1 and E_c_µ2 to values greater than zero (E_c_µ1>0 and E_c_µ2>0). The centre movie and graph (red line) depict alterations to G-actin distribution occurring following activation of actin polymerisation (E_c_µ1>0), but not cofilin phosphorylation (E_c_µ2=0). The right-hand movie and blue line in the graph depict predicted G-actin dynamics following activation of cofilin phosphorylation (E_c_µ2>0), but not actin polymerisation (E_c_µ1=0).

**Legends to supplementary data**

**Supplementary data 1: Proteomic analysis of protein translocation from the cell surface to the nuclear fraction**

*Sheet 1, the HGF-dependent cell surface to nucleus proteome:* H1299 cells were SILAC-labelled with medium or light amino acids and surface-labelled with NHS-SS-Biotin at 4°C. Cells were warmed to 37°C in the presence (light labelled cells; L) or absence heavy labelled cells; H) of HGF. Following this, nuclei were purified, lysed and biotinylated proteins isolated from these using streptavidin beads. Isolated proteins were analysed using mass spectrometry-based proteomics, analysed using Maxquant to obtain the SILAC ratios and these are presented in sheet 1 (nuc_hgf_nuc_ctrl) . The ratio of HGF-treated (L; Int Nucl HGF) to untreated (L; Int Nuc Ctrl) from two independent experiments (1 and 2) are presented in columns D and E respectively and ranked in descending order. The proteins in red are those which are represented in the surface proteome (see Sheet 2) and, therefore, have been categorized as moving from the plasma membrane to the nucleus fraction. These proteins are the ones denoted by red dots in figure 1b.

*Sheet 2, the surface proteome:* H1299 cells were SILAC labelled with heavy or light amino acids and surface-labelled with NHS-SS-Biotin. Light-labelled cells (L) were treated with MesNa to remove biotin from the cell surface, whilst heavy-labelled cells were left untreated (H). Cells were lysed, biotinylated proteins were isolated and analysed using mass spectrometry-based proteomics as above. The ratio of surface-reduced (L) to not-reduced (H) are presented in column D and ranked in ascending order. Thus, the proteins most enriched at the cell surface at the beginning of the experiment have the most negative values and are listed towards the top of column D in a blue typeface. These proteins are then denoted in red in sheet 1.

**Supplementary data 2: Proteomic analysis of the NLS-dependent proximity interactome of EphA2**

H1299 cells stably expressing TurboID fused to either wild-type (WT) EphA2 or NLS1/NLS2 EphA2 mutants were incubated with biotin and HGF for 1h at 37°C. After incubation, cells were lysed, and biotinylated proteins purified from the lysates using streptavidin beads. Purified proteins were eluted from the beads and subjected to label-free MS-based proteomics followed by analysis using MaxQuant software. LFQ intensity values were determined for proteins biotinylated in the presence of HGF by TurboID-EphA2 wild-type (LFQ intensity WT; columns J-N), TurboID-EphA2-NLS1 (LFQ intensity NLS1; columns O-S) and TurboID-EphA2-NLS2 (LFQ intensity NLS2; columns T-X). LQF values are from 5 independent experiments (denoted n=1, n=2, n=3, n=4 and n=5). Differences between the mean LFQ intensities of proteins biotinylated in the presence of HGF by EphA2 WT-TurboID-expressing cells and those from cells expressing TurboID-EphA2-NLS1 (Student’s t-test Difference WT versus NLS1; column E) or TurboID-EphA2-NLS1 (Student’s t-test Difference WT versus NLS2; column F) were calculated and ranked in descending order. The student’s t-test Difference values corresponding to nuclear pore components and related proteins are represented with coloured dots and annotations in Fig. 3c, whilst the Difference values for other (non-nuclear pore) proteins present in this dataset are represented by grey dots.

**Supplementary data 3: RNAseq analysis of HGF-dependent** **gene expression in the presence and absence of EphA2 knockdown.**

*Sheets 1 & 2, mRNAs upregulated by HGF in control and EphA2 knockdown cells:* H1299 cells were transfected with siRNAs targeting EphA2 (siEphA2; sheet 2) or a non-targeting control (siNT; sheet 1). 48hr following transfection, cells were challenged with HGF (+HGF) or vehicle control (-HGF) for 20 min. RNA was extracted and analysed by RNAseq using the Illumina Next Seq 500 system, followed by alignment to the GRCm38 version of the human genome and annotation using HiSat2 version 2.1.0. Expression levels were determined and statistically analysed by a combination of HTSeq version 0.9.1, the R environment version 3.4.4, utilizing packages from the Bioconductor data analysis suite and differential gene expression analysis was performed using voom pipeline from the limma package in R. HGF driven changes in mRNA level were calculated as the logarithm (Log2) of the ratio (logFC) of mRNA levels in the presence and absence of HGF (-HGF vs +HGF), and these are presented in for control (siNT; sheet 1) and EphA2 knockdown (siEphA2; sheet 2) cells. Data are ranked according to the logFC in descending order (column C).

*Sheet 3, HGF-driven mRNAs which are sensitive to EphA2 knockdown:* mRNAs which were differentially regulated by HGF following EphA2 knockdown were identified. These are the mRNAs indicated by the red dots and blue text in the upper left quadrant of the graph in figure 2. This sheet displays the values yielded by control (siNT) or EphA2 (siEphA2) knockdown cells incubated in the absence (-HGF) or presence (+HGF) of HGF from 3 individual experiments (1-3). These individual values are the ones represented in the heat map presented in figure 2d.
