# Supplementary material for: Nuclear-capture of endosomes depletes nuclear G-actin to promote SRF/MRTF activation and cancer cell invasion
Source: Nat Commun. 2021 Nov 24;12:6829. doi: 10.1038/s41467-021-26839-y (PMC8613289; doi:10.1038/s41467-021-26839-y)
Supplement: Supplementary file 14 — Reporting Summary [file 41467_2021_26839_MOESM14_ESM.pdf]

nature portfolio

Corresponding author(s): Prof. Jim C. Norman

Last updated by author(s): Oct 4, 2021

Reporting Summary

Nature Portfolio wishes to improve the reproducibility of the work that we publish. This form provides structure for consistency and transparency in reporting. For further information on Nature Portfolio policies, see our Editorial Policies and the Editorial Policy Checklist.

Statistics

For all statistical analyses, confirm that the following items are present in the figure legend, table legend, main text, or Methods section.

n/a	Confirmed	
☐	☒	The exact sample size (n) for each experimental group/condition, given as a discrete number and unit of measurement
☐	☒	A statement on whether measurements were taken from distinct samples or whether the same sample was measured repeatedly
☐	☒	The statistical test(s) used AND whether they are one- or two-sided<br>*Only common tests should be described solely by name; describe more complex techniques in the Methods section.*
☐	☒	A description of all covariates tested
☐	☒	A description of any assumptions or corrections, such as tests of normality and adjustment for multiple comparisons
☐	☒	A full description of the statistical parameters including central tendency (e.g. means) or other basic estimates (e.g. regression coefficient) AND variation (e.g. standard deviation) or associated estimates of uncertainty (e.g. confidence intervals)
☐	☒	For null hypothesis testing, the test statistic (e.g. F, t, r) with confidence intervals, effect sizes, degrees of freedom and P value noted<br>*Give P values as exact values whenever suitable.*
☒	☐	For Bayesian analysis, information on the choice of priors and Markov chain Monte Carlo settings
☒	☐	For hierarchical and complex designs, identification of the appropriate level for tests and full reporting of outcomes
☒	☐	Estimates of effect sizes (e.g. Cohen's d, Pearson's r), indicating how they were calculated

Our web collection on statistics for biologists contains articles on many of the points above.

Software and code

Policy information about availability of computer code

Data collection

To collect Airyscan confocal microscopy images Zeiss Black software version 2.3.sp1 was used (Zeiss). Mass spectrometry data was acquired using the XCalibur software (Thermo Fisher Scientific - Version 4.2.28.12). To acquire ELISA absorbance data, Magellan software version 7.2 (Tecan) was used. To obtain Western blotting data, image studio software version 5.2 was used (Li-Cor); and for ECL blots, Bio-Rad Chemidoc ImageLab Touch Software (version 2.3.0.07) was used. To obtain images for high-content analysis Harmony High-content Imaging and analysis software was used (version 4.9 - Perkin Elmer), and Columbus Image Data Storage and Analysis System (PerkinElmer - version 2.8.0). To obtain qPCR raw data Bio-Rad CFX Manager 3.1 software was used (BioRad Laboratories). To obtain time-lapse images, Metamorph software (version 7.8.13.0) was used (Molecular Devices). To obtain flow cytometry data, BD FACSDIVA software (version 8.0.1) was used.

Data analysis

For the mathematical model (Fig. 7a,c and supplementary Fig. 5d) simulations were performed in the Virtual Cell modelling and simulation software environment using the built-in Semi-Implicit Finite Volume-Particle Hybrid (regular grid, fixed time step) solver (see methods).

Mass spectrometry data was plotted using Perseus software version 1.6.0.7 and 1.6.2.3. For mass spectrometry SILAC data (Fig. 1b), raw data files were processed with MaxQuant software version 1.5.5.1 (Cox and Mann, 2008), and for BioID data (Fig. 3c) with MaxQuant version 1.6.14.0, and then searched using the Andromeda search engine (Cox et al., 2011) against the human UniProt database (09/07/2016; 92.939 entries). Enrichment plots were generated using GSEA 4.1.0 software (Broad Institute).

To analyse RNAseq expression data (Fig. 5a), quality checks on the raw RNASeq data files were performed using fastqc version 0.11.7 and fastq screen version 0.12.0. RNAseq paired-end reads were aligned to the GRCm38 version of the human genome and annotation using HiSat2 version 2.1.0. Expression levels were determined and statistically analysed using a combination of HTSeq version 0.9.1, the R environment version 3.4.4, utilizing packages from the Bioconductor data analysis suite and differential gene expression analysis was performed using voom pipeline from the limma package in R (see methods).

To analyse qPCR raw data, BioRad CFX Manager 3.1 software was used throughout (BioRad Laboratories).

To analyse microscopy images FIJI/ImageJ sofware was used throughout (version1.53).

For high-content image analysis (Fig. 4c; 6d) Columbus Image Data Storage and Analysis System software version 2.8.0 was used (Perkin Elmer).

To analyse flow cytometry data (Fig. 6c), FlowJo software version 10.1r5 was used (FlowJo, LLC).

For manuscripts utilizing custom algorithms or software that are central to the research but not yet described in published literature, software must be made available to editors and reviewers. We strongly encourage code deposition in a community repository (e.g. GitHub). See the Nature Portfolio guidelines for submitting code & software for further information.

Data

Policy information about availability of data

All manuscripts must include a data availability statement. This statement should provide the following information, where applicable:

- Accession codes, unique identifiers, or web links for publicly available datasets
- A description of any restrictions on data availability
- For clinical datasets or third party data, please ensure that the statement adheres to our policy

The SILAC Nuclear Capture Proteome data generated in this study have been deposited in the ProteomeXchange Consortium via the PRIDE partner repository under the accession code PXD027268. The RNA sequencing data generated in this study have been deposited in the GEO – NCBI – NIH repository under the accession number GSE179901 (https://www.ncbi.nlm.nih.gov/geo/query/acc.cgi?acc=GSE179901). The EphA2 TurboID proteomic data generated in this study have been deposited in the ProteomeXchange Consortium via the PRIDE partner repository under the accession code PXD027217.
The remaining data are available within the Article, Supplementary Information. All raw data were generated at the CRUK Beatson Institute. All raw data and data derived from this which are relevant to this study are available from the corresponding author [JCN] on request.

Field-specific reporting

Please select the one below that is the best fit for your research. If you are not sure, read the appropriate sections before making your selection.

☒ Life sciences ☐ Behavioural & social sciences ☐ Ecological, evolutionary & environmental sciences

For a reference copy of the document with all sections, see nature.com/documents/nr-reporting-summary-flat.pdf

Life sciences study design

All studies must disclose on these points even when the disclosure is negative.

Sample size	Sample sizes consisted at least of three biological repeats per experiment. The sample sizes were not pre-determined as most of the experiments showed a clear reproducibility after three biological repeats. If a technique/experiment displayed a clear and robust trend, but the intrinsic variability of the technique did not demonstrate statistical significance after three repeats, then the number of independent repeats was be increased to ensure that variability was correctly controlled for.
Data exclusions	No data exclusions apply for the analysis of the experiments performed.
Replication	Each individual experiment was repeated independently at least three times. All attempts at replication were successful and experiments were only discarded when technical issues precluded interpretation (i.e. an antibody failing to work or qPCR cycles too low, etc.).
Randomization	No randomisation was applied to the experiments performed. The use of cell lines in this study allowed for no randomisation since the homogeneity of the samples allows for a high degree of confidence that possible covariates are controlled.
Blinding	Blinding was not employed. The quantification applied for cell biological experiments was not selective or subjective, therefore, it did not require blinding.

Reporting for specific materials, systems and methods

We require information from authors about some types of materials, experimental systems and methods used in many studies. Here, indicate whether each material, system or method listed is relevant to your study. If you are not sure if a list item applies to your research, read the appropriate section before selecting a response.

Materials & experimental systems

n/a	Involved in the study
☐	☒ Antibodies
☐	☒ Eukaryotic cell lines
☒	☐ Palaeontology and archaeology
☒	☐ Animals and other organisms
☒	☐ Human research participants
☒	☐ Clinical data
☒	☐ Dual use research of concern

Methods

n/a	Involved in the study
☒	☐ ChIP-seq
☐	☒ Flow cytometry
☒	☐ MRI-based neuroimaging

Antibodies

Antibodies used

For Western blotting and immunofluorescence, antibodies were from the following sources: goat anti-LaminA/C (Santa Cruz, sc-6215), mouse anti-EphA2 (Millipore, 05-480), mouse anti-EphA2 (Santa Cruz, sc-398832), rabbit anti-EphA2 phospho-Ser897 (Cell Signaling, #6347), rabbit anti-EphA2 phospho-Tyr588 (Cell Signaling, #12677), mouse anti-GFP (Immunoprecipitations, Abcam, ab1218), mouse anti-GFP (Western Blot, Santa Cruz, sc-9996), rabbit anti-GFP (Abcam, ab6556), rabbit anti-importin-α5 (KPNA1, Proteintech, 18137-1-AP), mouse anti-importin-β1 (KPNB1, Cell Signaling, #60769), mouse anti-actin (Sigma, A1978), mouse anti-RhoG (Millipore, 04-486), mouse anti-Rab17 (Abnova, H00064284-MO1), mouse anti-importin-α7 (Proteintech, 12366-2-AP), rabbit anti-TNPO3 (Abcam, ab109386), mouse anti-CHC (TD1, gift from F. Brodsky), rabbit anti-HistoneH2a (Abcam, ab16563) and rabbit anti-XPO6 (Proteintech, 11408-1-AP), mouse anti-cofilin (Proteintech, 66057-1-Ig) and rabbit anti-cofilin phospho-Ser3 (Santa Cruz, sc-12912-R). For immunoprecipitation, mouse antibodies were coupled to magnetic beads conjugated to anti-mouse IgG (Invitrogen; Dynabeads Sheep anti-mouse IgG; catalogue number 11031). For chromatin immunoprecipitation, antibodies were rabbit anti-SRF (Cell Signaling Technologies, #5147), rabbit anti-MRTF-A (Cell Signaling Technologies, #14760) and mouse anti-ELK-1 (Santa Cruz, sc-365876). For the TurboID experiments, we used mouse anti-RanBP2 (Santa Cruz, sc-74518), rabbit anti-Nup214 (Bethyl Laboratories, A300-716A-M), rabbit anti-POM121 (Genetex, GTX102128), mouse anti-Nup133 (Santa Cruz, sc-376763), mouse anti-Nup88 (BD Biosciences, 611896), mouse anti-Nup50 (Santa Cruz, sc-398993), goat anti-laminA/C (Santa Cruz, sc-6215) and mouse anti-V5-Tag (Thermo, MA5-15253).

Validation

Where possible the antibodies were tested using siRNA silencing approaches together with either Western blotting or immunofluorescence staining, coupled with qPCR to test the efficiency of the silencing. Furthermore, antibodies were checked by determining whether they were gave rise to bands in the expected molecular weight on Western blotting.

For calnexin, syntaxin-6, tubulin, histone H2a antibodies were checked by Western blotting for appropriate molecular weight and also for cellular location using cell frationation (fig.1c); streptavidin-Alexa488 was validated using NHS-SS-Biotin labelled samples from which the biotin tag had been removed by reduction with beta-mercaptoethanol (fig.1a); clathrin antibody was validated by using a combination of siRNA and Western blotting (fig.2a); Rab17 antibody was assessed using siRNA and Western blotting (Fig. 2e); RanBP2 antibody was checked by siRNA and Western blotting (supplementary fig.3e); Nup214, POM121, Nup133, Nup88, Nup50 were assessed by confirming the molecular weight of the bands visible by Western blotting (Fig. 3e); importin alpha-5 and importin beta-1 were assessed by observing their molecular weight on Western blotting (Fig. 3f); EphA2 antibody was validated using mouse cells which were knockout for EphA2, and in human cells using siRNA combined with Western blotting (Fig.4a and d); cofilin, phospho cofilin and actin antibodies were validated by expected molecular weight in Western blotting (Fig.7b); V5tag antibody was validated by immunofluorescence in non-transfected cells (supplementary Fig.1b); importin alpha-7 and transportin-3 were validated by observation of the expected molecular weight by Western blotting (supplementary Fig.1e); EphA2 phospoSer897 antibody has been previously validated by our laboratory both by immunofluorescence and Western blotting combined with siRNA (Gundry et al. 2017 Nat Commun - https://doi.org/10.1038/ncomms14646); the GFP antibody was validated by Western blotting in non-transfected cells (Fig.4a and d), RhoG antibody was validated by Western blotting from cells expressing exogenous RhoG-GFP (supplementary Fig.4a), exportin-6 was validated by siRNA combined with Western blotting (supplementary Fig.4f). Finally, the lamin A/C antibody was validated by immunofluorescence (Fig. 1a).

Eukaryotic cell lines

Policy information about cell lines

Cell line source(s)	H1299 (NCI-H1299, ATCC); KPC (KRas, P53 mutant, Pdx-Cre) (generated in-house from mice tumours).
Authentication	The genetic identity of all these cell lines was confirmed at the CRUK Beatson Institute (Promega GenePrint 10 Kit).
Mycoplasma contamination	All cell lines tested negative for mycoplasma.
Commonly misidentified lines (See ICLAC register)	No commonly misidentified cell lines were used in this study.

Flow Cytometry

Plots

Confirm that:

- [x] The axis labels state the marker and fluorochrome used (e.g. CD4-FITC).
- [x] The axis scales are clearly visible. Include numbers along axes only for bottom left plot of group (a 'group' is an analysis of identical markers).
- [x] All plots are contour plots with outliers or pseudocolor plots.
- [x] A numerical value for number of cells or percentage (with statistics) is provided.

Methodology

Sample preparation	Purified nuclei were obtained from H1299 cells. Nuclei were fixed with 4% paraformaldehyde, permebilised with 0.2% triton x-100/PBS, and stained with DAPI and DNAseI-Alexa594 for 1 hour at room temperature. At least 10.000 events per condition per experiment were measured in the cytometer.
Instrument	BD LSRFortessa
Software	FlowJo (FlowJo, LLC - 10.1r5)
Cell population abundance	Purified nuclei, and not intact cells were analyzed by flow cytometry in this paper, therefore this parameter is not applicable. here.
Gating strategy	Purified nuclei were gated according to FSC and SSC. DAPI positive events were selected. Alexa-594 fluorescence was analysed in this population.

- [x] Tick this box to confirm that a figure exemplifying the gating strategy is provided in the Supplementary Information.
